# Chitosan in Molecularly-Imprinted Polymers: Current and Future Prospects

**DOI:** 10.3390/ijms160818328

**Published:** 2015-08-07

**Authors:** Long Xu, Yun-An Huang, Qiu-Jin Zhu, Chun Ye

**Affiliations:** 1 School of Liquor & Food Engineering, Guizhou University, Guiyang 550025, China; E-Mails: xulong19891026@163.com (L.X.); hya127@163.com (Y.-A.H.); yechun126@163.com (C.Y.); 2 Key Laboratory of Agricultural & Animal Products Store and Processing of Guizhou Province, Guiyang 550025, China

**Keywords:** chitosan, molecularly imprinted polymers, cross-linkers, aldehydes, heterocyclic compounds

## Abstract

Chitosan is widely used in molecular imprinting technology (MIT) as a functional monomer or supporting matrix because of its low cost and high contents of amino and hydroxyl functional groups. The various excellent properties of chitosan, which include nontoxicity, biodegradability, biocompatibility, and attractive physical and mechanical performances, make chitosan a promising alternative to conventional functional monomers. Recently, chitosan molecularly-imprinted polymers have gained considerable attention and showed significant potential in many fields, such as curbing environmental pollution, medicine, protein separation and identification, and chiral-compound separation. These extensive applications are due to the polymers’ desired selectivity, physical robustness, and thermal stability, as well as their low cost and easy preparation. Cross-linkers, which fix the functional groups of chitosan around imprinted molecules, play an important role in chitosan molecularly-imprinted polymers. This review summarizes the important cross-linkers of chitosan molecularly-imprinted polymers and illustrates the cross-linking mechanism of chitosan and cross-linkers based on the two glucosamine units. Finally, some significant attempts to further develop the application of chitosan in MIT are proposed.

## 1. Introduction

**Figure 1:**
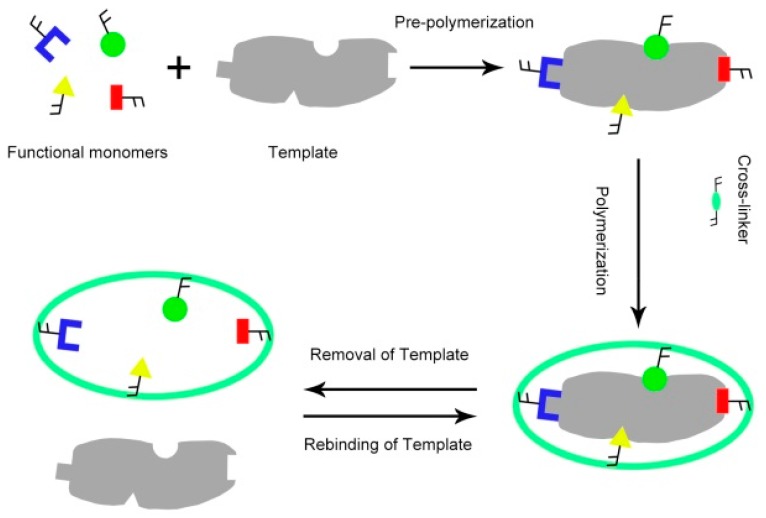
Schematic of molecular imprinting.

Molecular imprinting is a process in which interacting and cross-linking monomers are arranged around a molecular template, followed by polymerization to form a cast-like shell. When first described in the 1970s and early 1980s by Wulff [1] and Mosbach [2], molecularly-imprinted polymers (MIPs) were merely used, for example, as specific separation materials for the chromatographic separation of enantiomers. In 1993, Mosbach’s seminal paper in Nature [3] revealed that the great potential of MIPs as synthetic antibody mimics was recognized. At present, MIPs have attracted considerable attention because of their outstanding advantages, which include predetermined recognition ability, stability, relative ease and low cost of preparation, and potential application in a wide range of target molecules [4,5,6]. These advantages result in a nearly exponential increase in the number of publications in the area, with several hundred papers per annum being released over the recent years. Initially, monomers form a complex with a template molecule through covalent or non-covalent interactions and are then joined by a cross-linking agent. After polymerization, removal of the template by chemical reaction or extraction exposes binding sites that are complementary to the target molecule in size, shape, and position of the functional groups, as shown in Figure 1. These binding sites are held in place by the cross-linked polymer matrix and consequently allow selective uptake. To date, MIPs have been successfully used as artificial receptors in separations [7,8], sensors [9], catalysis [10], drug development and screening [11], solid-phase extraction [12,13], and chromatographic separation [14].

**Figure 2:**
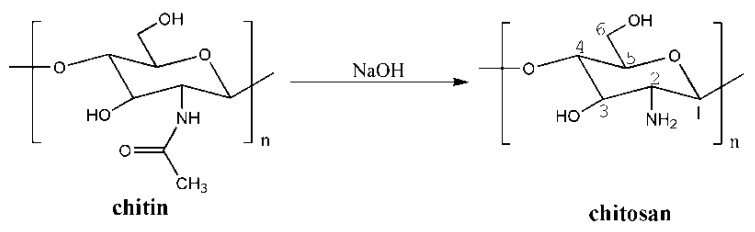
Deacetylation of chitin.

Chitosan is a type of natural polyamino-saccharide that is synthesized from the deacetylation of chitin, which is a polysaccharide that consists predominantly of unbranched chains of β-(1→4)-2-acetoamido-2-deoxy-d-glucose (Figure 2) [15]. This material has been considered as one of the most promising biopolymers for the development of advanced materials because of its excellent properties, such as abundance, nontoxicity, biodegradability, and biocompatibility. However, chitosan’s poor physical properties and high swelling degree in aqueous systems limit its practical application [16]. At low pH, although protonation of the amino groups of chitosan and its composite facilitate the absorption of heavy metal ions, dyes, and protein molecules via various interaction mechanisms, including electrostatic attractions and chelation, glycosidic bonds of chitosan may be hydrolyzed so as to lead to loss by dissolution and poor stability. Separation of specific ions or compounds by chitosan is unfeasible.

As an effective method of preparing chitosan MIPs, chemical cross-linking is conventionally used to overcome the aforementioned shortcomings. The active amine and hydroxyl groups on the polysaccharide chain enable structural modification and cross-linking in MIP preparation. In chitosan MIPs, chitosan can perform not only as a functional monomer but as a supporting matrix. Moreover, given the stronger electronegativity of oxygen in comparison with that of nitrogen, –OH is more likely to participate in nucleophilic reaction. Furthermore, compared with OH–C_3_, OH–C_6_ is more likely to participate in nucleophilic reaction because of its weaker steric effects. The conventional preparation of chitosan MIPs has involved numerous cross-linkers including glutaraldehyde [17], epichlorohydrin [18], sulfuric acid [19], and glyoxal [20]. These cross-linkers can not only stabilize chitosan but also enhance its mechanical properties [21]. In the present review, we focus on the current status of the commonly used cross-linkers in chitosan MIPs (Table 1), and then illustrate the cross-linking mechanism of chitosan and its cross-linkers. Based on the current work and extensive investigation in our laboratory, we also attempt to explore the future development direction of chitosan MIPs.

## 2. Performance of Cross-Linkers in MIPs

Cross-linkers in polymeric networks secure the functional groups of functional monomers in specific locations and directions around template molecules to preserve the structure of the binding site (cavity) [22]. After elution of the template, the formed holes should be completely complementary to the target molecules in terms of shape and functional groups. The structure of the imprinted cavities should be stable enough to maintain the conformation in the absence of the template but should remain sufficiently flexible to facilitate the attainment of a rapid equilibrium between the release and re-uptake of the template in the cavity. In addition, the cross-linker is important in controlling the morphology of the polymer matrix, whether gel-type, macroporous or a microgel powder in form [23]. In addition, numerous studies have shown that cross-linking degree would influence polymer properties, including particle size, rigidity of the cavity structure, flexibility of the polymer, and so on. Mostly imprinted systems for analytical applications require around 70% to 90% of cross-linking degrees [24]. Cross-linking levels could increase the hydrophobicity of the network and prevent the polymer network from changing the conformation obtained during synthesis. As a consequence, the affinity for the template is not dependent on external variables. Moreover, the amounts and types of cross-linkers exert profound influences on the selectivity and binding capacity of MIPs. A low dosage of cross-linkers will not allow MIPs to maintain stable cavity configurations because of the low cross-linking degree. However, excessively high amounts of cross-linkers will considerably increase the stiffness of the network, as well as reduce the number of recognition sites per unit mass of MIPs. Furthermore, in order to achieve good imprinting effects, the chemical reactivity of the cross-linker should be kept similar to that of functional monomers. Otherwise, the monomers or cross-linkers will dominate during polymerization, which can lead to inadequate copolymerization reaction. Doehlert’s experimental design, which is a second-order uniform shell design, has been proposed for the optimization of the nature and amount of the cross-linker and its influence on template recognition [25].

## 3. Crosslinking Effect of Different Categories of Compounds

### 3.1. Aldehydes

**Figure 3:**
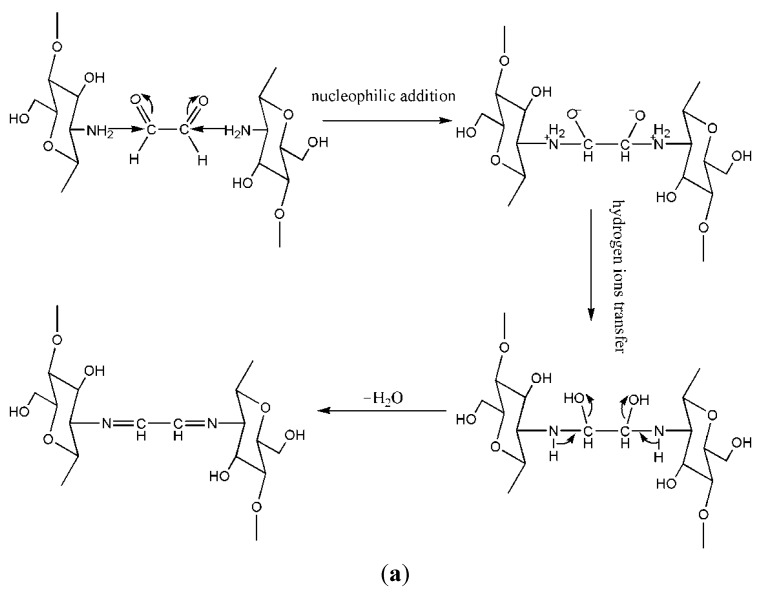

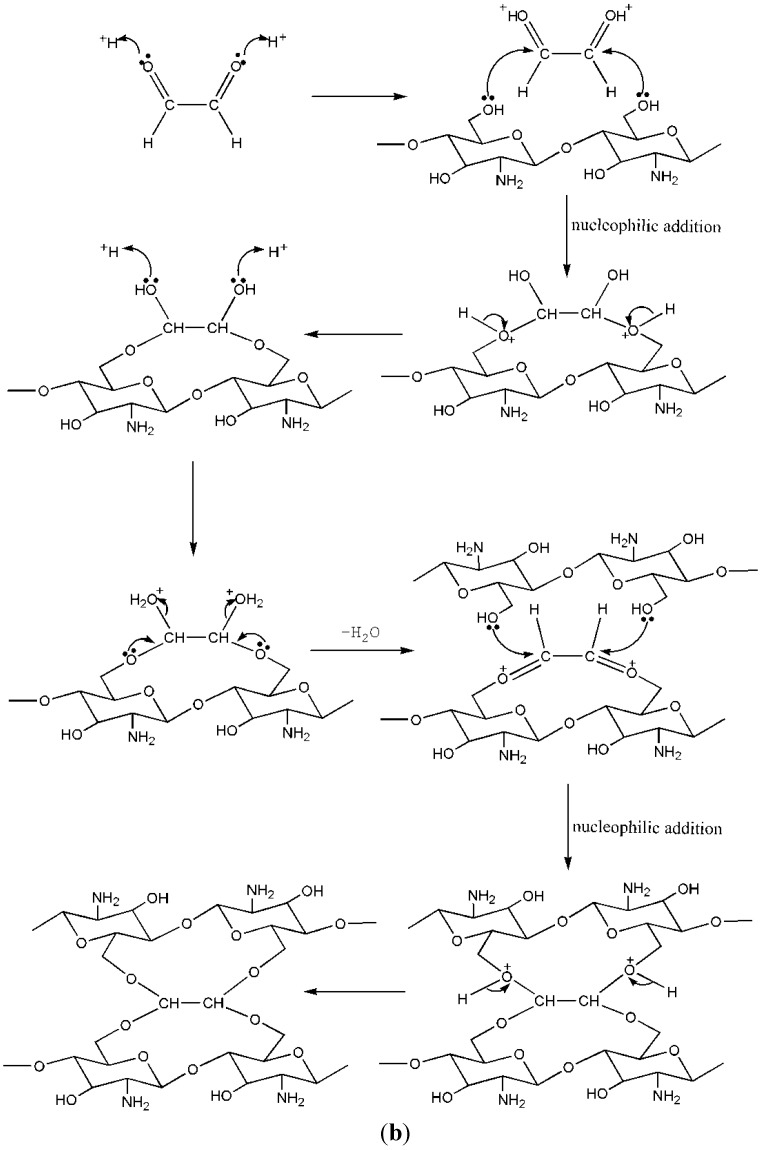
(**a**) Mechanism of primary reaction of chitosan and glyoxal; (**b**) Mechanism of secondary reaction of chitosan and glyoxal.

Chitosan can easily cross-link with dialdehydes. Cross-linked chitosan derivatives usually show stronger hydrophobic properties. This type of cross-linker mainly contains glyoxal and glutaraldehyde. Numerous properties of chitosan, including hygroscopicity, ion permeability, and mechanical property, will be markedly changed after being cross-linked by dialdehydes [26]. For example, glyoxal, which is one of the bifunctional coupling agents with two highly reactive aldehyde groups, is an efficient cross-linker because of its cross-linking ability via acetal formation between the hydroxyl groups of chitosan glucosamine units and the aldehyde groups of glyoxal, or through Schiff’s base formation between the unsubstituted free amino groups of chitosan and the aldehyde groups of glyoxal [27]. Numerous previous studies have been reported regarding the improvement of the mechanical properties of chitosan through glyoxal cross-linking for various biomedical applications [28,29]. The mechanism of the primary reaction and secondary reaction of chitosan and glyoxal are presented in Figure 3.

Monier *et al.* [30] prepared an enantioselective l-aspartic acid imprinted chitosan (LAIC) by a glyoxal cross-linker and, in the presence of l-aspartic acid, as an imprinted template molecule and, in 1% acetic acid solution, as a solvent. Non-imprinted cross-linked chitosan (NIC) was also prepared as control by using the same procedure, but in the absence of template molecules. In their study, they demonstrated two types of cross-linking mechanisms between chitosan and glyoxal. The occurrence of acetalization and Schiff base reaction plays an important role in cross-linking reaction. Adsorption isotherms indicate that the maximum adsorption capacities of l- and d-aspartic acid on LAIC were (48 ± 0.7) and (27 ± 1) mg/g, respectively, whereas, in the case of NIC, both l- and d-aspartic acid present the same maximum adsorption capacity of (9 ± 0.8) mg/g. And they pointed out that the majority of the adsorption was due to the protonated C=N active sites, which are predominant in the cross-linked network. Liu *et al.* [31] successfully synthesized a novel bio-based α-Fe_2_O_3_ impregnated chitosan beads (As-IFICB) using As(III) as imprinted ions and glutaraldehyde as cross-linker for adsorption and removal of As(III) ions from aqueous solutions. The results showed that the maximum adsorption capacity was 6.18 mg/g at pH = 5, 30 °C. The selectivity coefficient of As(III) ions and other metal cations onto As-IFICB indicate an overall preference for As(III) ions. This preference was markedly higher than that of non-imprinted chitosan beads. The synthesis and application As-IFICB as an alternative adsorbent in the separation, preconcentration, and extraction of As(III) from aqueous solutions presents a promising method for researchers. This cross-linking modification can not only increase the surface area but reinforce the chemical strength of MIPs in acidic medium. Liu *et al.* [32] prepared a Cd(II)-imprinted chitosan resin (Cd-ICR) by using glutaraldehyde as a cross-linker for the adsorption of Cd(II) from oyster hydrolysate and aqueous solutions. Fourier Transform Infrared Spectroscopy analysis indicated that the amino group and secondary hydroxyl group played a vital role in the Cd-ICR chelation process. Batch adsorption experiments indicate that the maximum adsorption capacity of Cd-ICR was 0.795 mmol/g in sole Cd(II) solution at pH 5.0, 45 °C, and with 10 h equilibrium time. The selectivity coefficient of Cd(II) and other metal cations on Cd-ICR indicated an overall preference for Cd(II), which was considerably higher than that of the non-imprinted chitosan resin. The removal ratio of Cd(II) from oyster hydrolysate was 73.6%, whereas those of Ca(II) and Zn(II) were 6.2% and 9.9%, respectively. This finding suggests that Cd-ICR is a highly promising adsorbent for the selective removal of Cd(II) from aqueous solutions. Above all, we can clearly see that chitosan MIPs cross-linked by dialdehydes especially glyoxal and glutaraldehyde can not only enhance the mechanical property, but stabilize the structure and keep the imprinting effect as well.

### 3.2. Heterocyclic Compounds

In addition to aldehyde cross-linkers, several heterocyclic compounds have also been reported to cross-link chitosan. In these heterocyclic compounds, other atoms including oxygen, sulfur, and nitrogen, can also be involved in cyclization besides carbon atoms. Heterocyclic compounds easily get their loops to open into chain compounds when used for cross-linking. These compounds mainly contain epichlorohydrin (ECH), (chloromethyl) thiirane, cycloolefin terpenoids (Genipin) [33,34], and several anhydrides (including maleic anhydride, and succinic anhydride) [35,36]. Part of the functional groups, like –OH and –COOH, will be introduced into the cross-linked polymers after the polymerization of chitosan and the cross-linker, so that the cross-linked product can combine with the template molecule. Genipin, which is low in toxicity but is too expensive, is conventionally used in medical materials and the food industry. In contrast, ECH and (chloromethyl) thiirane are of high toxicities. Reaction conditions must be strictly controlled when anhydrides are used as cross-linkers to avoid the dissolution of chitosan because of *N*-acetylation. Therefore, the selection of this type of cross-linker depends on their purpose. At present, genipin and, particularly, ECH have been reported in the synthesis of chitosan MIPs.

#### 3.2.1. Epichlorohydrin (ECH)

**Figure 4:**
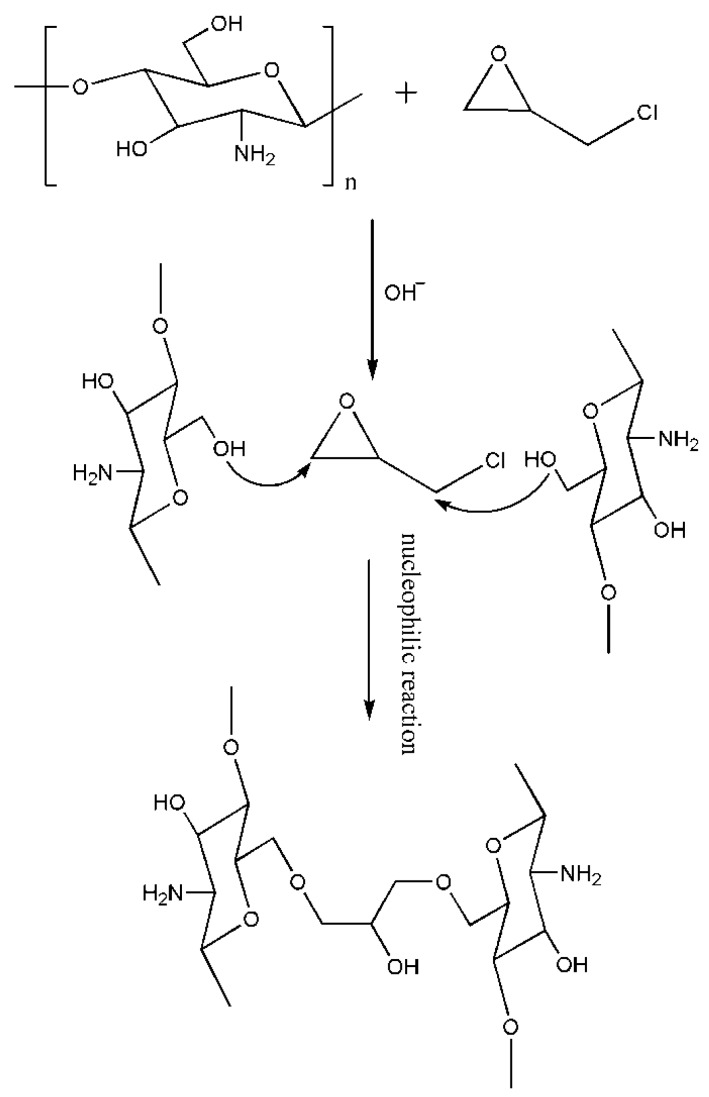
Cross-linking mechanism of chitosan and ECH under alkaline conditions.

Chemical cross-linking with ECH is a basic method of producing chitosan-based polymers. This cross-linking agent has been known for past 50 years and is relatively easy to use. ECH is the most common cross-linker used in polysaccharide chemistry [37]. This bi-functional agent comprises two reactive functional groups, namely, an epoxide group and a chloroalkyl moiety, which can form bonds with the –OH and –NH_2_ groups of chitosan. An aqueous solution of formaldehyde is usually involved in the cross-linking reaction of chitosan and ECH, functioning as an amino protective solute to prevent the cross-linking of ECH with –NH_2_. In particular, formaldehyde can protect –NH_2_ from protonation so as to avoid dissolution losses and improve the stability of chitosan. The cross-linking mechanism of chitosan and ECH is presented in Figure 4.

Chen *et al.* [38] prepared a novel type of adsorbent called thiourea-modified magnetic ion-imprinted chitosan/TiO_2_ (MICT) by using ECH as a cross-linking agent for grafting of sulfur groups through the combination of ion-imprinted technology and photodegradation technology. In addition, the group investigated the simultaneous adsorption of cadmium ion and 2,4-dichlorophenol (2,4-DCP) degradation by this novel composite adsorbent. In this MICT, the amino and hydroxyl groups are the major functional groups involved in cadmium bonding. The maximum adsorption capacity of cadmium was 256.41 mg/g according to the Langmuir model, and the 2,4-DCP degradation efficiency was up to 98% at the initial 2,4-DCP concentration of 10 mg/L. The adsorbed cadmium ion can be desorbed easily, and the sorption and degradation capacities are minimally affected by the number of cycles. The results obtained in the present study show that the MICT composite is an ideal platform to accelerate the simultaneous disposal of heavy metals and organic pollutants in wastewater. Huo *et al.* [39] prepared a Ag^+^-imprinted biosorbent for the treatment of wastewater contaminated with Ag^+^ by using ECH as a cross-linking agent and by surface MIT. The imprinted biosorbent showed higher adsorption affinity and selectivity toward the imprinting ion (Ag^+^) in comparison with the other non-imprinting metal ions. The maximum adsorption capacity was 199.2 mg/g at an initial Ag^+^ concentration of 1200 mg/L and biosorbent dosage of 3.0 g/L. The surface Ag^+^-imprinted biosorbent offered the advantages of low chitosan dosage and reduction of cost, and was predicted to be applied in the treatment and recovery of silver-bearing wastewater from electroplating, coinage, photo fixation, and so on. Yu *et al.* [40] prepared a novel chitosan-based MIP by cross-linking with ECH in the presence of perfluorooctane sulfonate (PFOS) as the template. The optimized MIP adsorbents exhibited a sorption amount of 560 mmol/g for PFOS, whereas the sorption amount of the NIP was only 258 mmol/g. ECH keeps the interactions between functional groups and enables the MIPs with conformation memory effectively. Electrostatic interaction performed a crucial function in recognizing the target compound during the sorption process. Sorption experimental results show that the prepared MIP adsorbents possessed high sorption capacity and good selectivity toward PFOS. The MIP adsorbents exhibited excellent regeneration performance and can be used for a minimum of five times without any loss of adsorption capacity toward PFOS. These results indicate the potential application of MIP adsorbents for the selective removal of PFOS in water or wastewater treatment. As we can see, chitosan MIPs cross-linked by ECH are normally used in ion imprinting. C_6_–OH is mainly involved in the cross-linking reaction in the presence of amino protective solute. The retained –NH_2_ played an important role in chelating ions during adsorption so as to enable chitosan ion-imprinted polymers with better adsorption efficiency.

#### 3.2.2. Genipin

**Figure 5:**
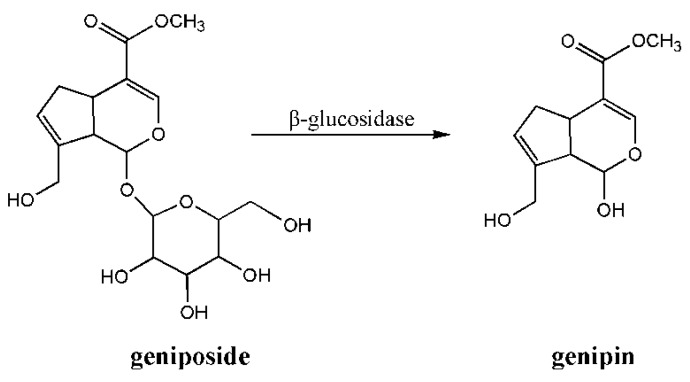
Chemical formulas for geniposide and genipin.

**Figure 6:**
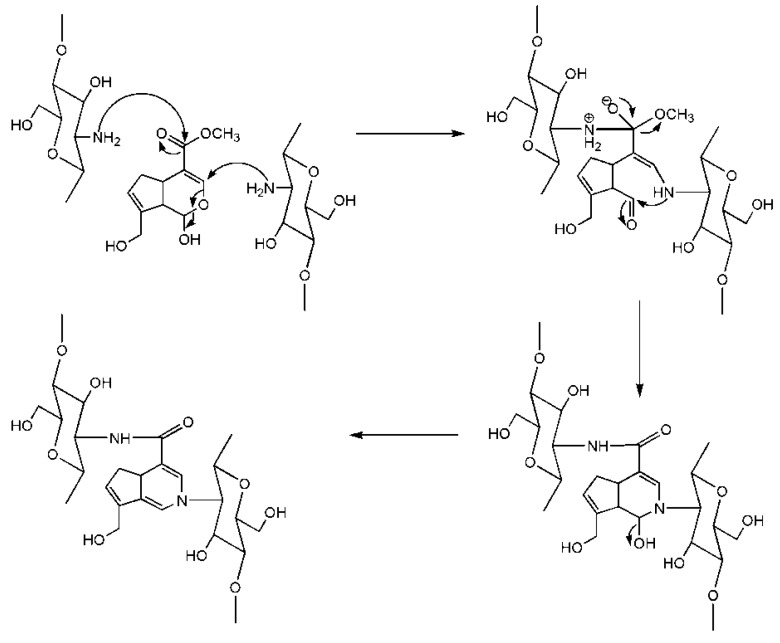
Cross-linking mechanism of chitosan and genipin.

Genipin is obtained from its parent compound, geniposide, via enzymatic hydrolysis with β-glucosidase. The formulas of genipin and geniposide are depicted in Figure 5. Genipin is a particularly nontoxic cross-linker and an effective, naturally-occurring cross-linking agent that can react spontaneously with amino acids or proteins to form dark blue pigments [41,42,43,44,45]. The cross-linking mechanism of chitosan and genipin is presented in Figure 6. Additionally, genipin could further associate to form short chains of cross-linking bridges. Bibiana *et al.* [46] prepared a molecularly-imprinted material from hydrogels of chitosan cross-linked with genipin by using *O*-xylene as a template molecule. Then the group investigated the gelling time, as well as the mechanical and diffusion properties of chitosan-genipin hydrogels. The adsorption capacity of MIP*_o_*_-xylene_ exceeded those of the corresponding control hydrogels. The imprinted hydrogel showed higher adsorption capacity for *O*-xylene than the *m*- and *p*-xylene isomers. The molecular recognition experiment demonstrated that binding sites were influenced by the electronic and steric properties of the analyte molecule, thereby effectively confirming the imprinting effect within the MIP*_o_*_-xylene_ network.

### 3.3. Ethers

This widely used type of cross-linker mainly contains several cyclic ethers, particularly ethylene glycol diglycidyl ether (EGDE) and crown ether which impart some excellent properties, such as the tension of multiple rings in the molecular structures, active chemical properties and easy opening of loops under acid or alkali catalysis. Yi *et al.* [47], Wan *et al.* [48] and Wang *et al.* [49] reported chitosan cross-linked by crown ether for metal ion adsorption. However, we did not find crown ether used as a cross-linker in chitosan MIPs. EGDE possesses two epoxide functional groups (cyclical ethers constituted by a three-membered ring) located at both ends of each molecule. EGDE is comparatively more reactive than other ethers because of the high energy associated with considerable strains that exist in the three-membered ring. The opening of the epoxide ring and the chemical interaction with amino, carboxyl, and hydroxyl functional groups can occur in acidic or in basic media [50,51].

**Figure 7:**
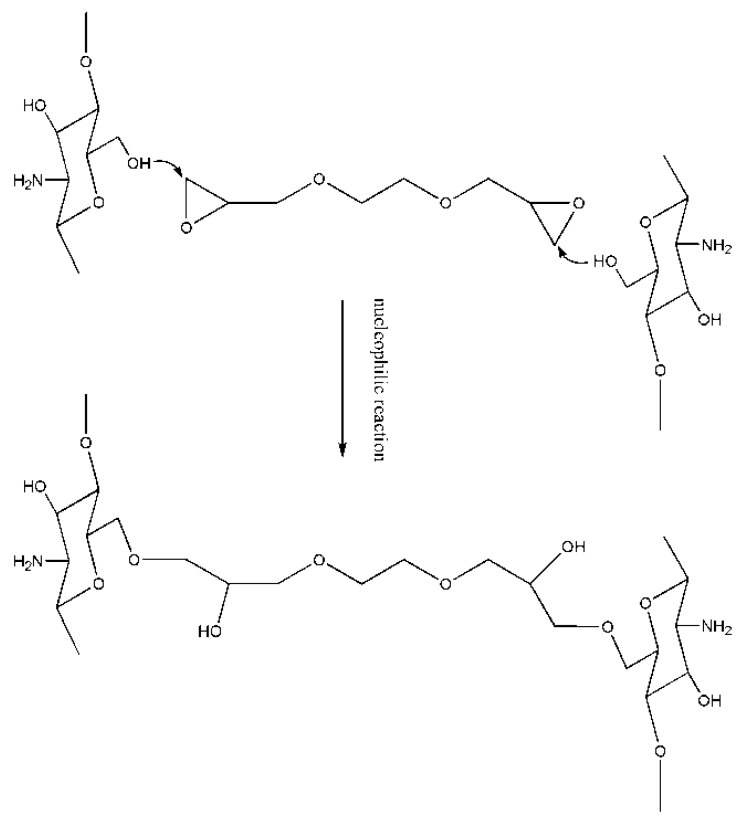
Cross-linking mechanism of chitosan and EGDE.

Similar to the mechanism involving ECH, the cross-linking mechanism of EGDE and chitosan, involves two types of ring-opening reactions. The open-loop epoxy group reacts with –NH_2_ or –OH of chitosan is mainly composed of two kinds of cross-linking reactions: (1) intra-molecular cross-linking reactions, in which the cross-linker reacts with two groups of one chain; and (2) inter-molecular cross-linking reactions, in which the cross-linker reacts with two groups of two chains. The hydrophilic group, –OH, will be introduced through the cross-linking reaction. However, the net structure renders the resultant polymer insoluble in water and dilute acid or dilute alkali. One cross-linking mechanism of chitosan and EGDE is presented in Figure 7.

Liu *et al.* [52] reported an interpenetration network (IPN) ion-imprinting hydrogel (IIH), which is composed of chitosan and polyvinyl alcohol (PVA), was synthesized using uranyl ions as a template and EGDE as a cross-linker for formation of UO_2_^2^^+^-imprinted hydrogel. Researchers illustrated three cross-linking structures between hydroxyl groups. The adsorption of uranyl ions on IIH followed the Langmuir and Freundlich isotherms. The kinetics of adsorption followed a pseudo-second order rate equation. The overall selectivity for uranyl ions showed that ion-imprinted chitosan/PVA cross-linked hydrogel can effectively remove uranyl ions from aqueous solutions. Tan *et al.* [18] reported a new method for the preparation of metal ion imprinted chitosan resin that can considerably enhance the adsorption capacity and selectivity of the metal ion. Among influencing conditions, the cross-linking agents, ECH and EGDE, was optimized. Cross-linked imprinted resins with EGDE exhibit good chemical and physical stability and can be used numerous times without losing its adsorption capacity. Adsorption isotherms can be described by Freundlich model. Moreover, researchers indicated that the adsorption capacity of metal ions is greatly decreased by glutaraldehyde cross-linking, because the cross-linking agent mainly reacts with –NH_2_ groups. As a result, the residual –NH_2_ functional groups are reduced, which thereby being disadvantageous for synthesizing chitosan-metal ion imprinted polymers.

### 3.4. Amides

**Figure 8:**
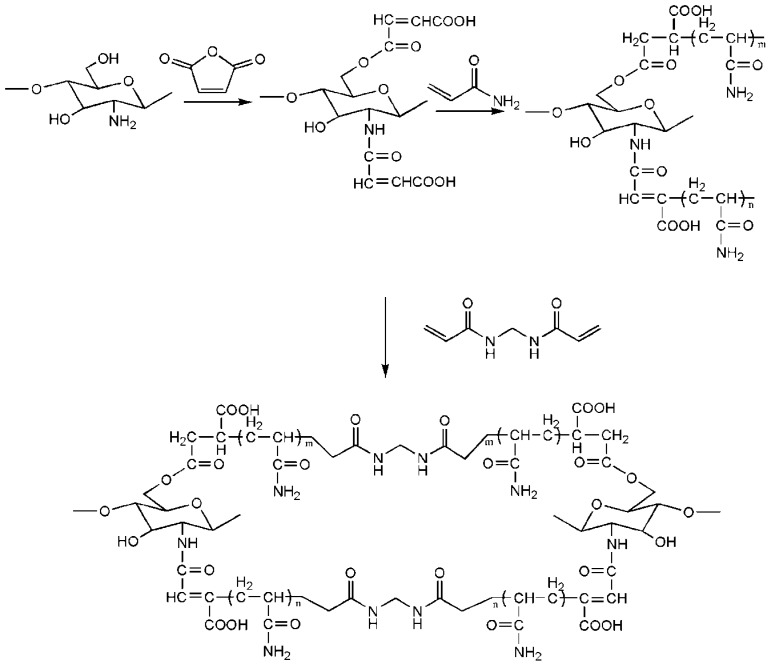
Cross-linking mechanism of maleic anhydride-modified chitosan and MBA.

In these types of cross-linkers, *N*,*N′*-methylenebisacrylamide (MBA) is frequently used in protein molecularly-imprinting technology. In chitosan-protein MIPs, chitosan or modified chitosan usually functions as a supporting matrix. Vinyl groups are usually introduced to chitosan beads for modification so that the functional monomer, including polyacrylamide or acrylamide, could be grafted to modified chitosan beads. These modifications were proposed to improve pore size, mechanical strength, chemical stability, hydrophilicity, and biocompatibility. Maleic anhydride is one of the widely used compounds for chitosan modification. In addition, aldehyde groups can also be introduced to chitosan beads, so that the template protein could be immobilized onto modified chitosan beads. The cross-linking mechanism of maleic anhydride-modified chitosan and MBA is presented in Figure 8.

Fu *et al.* [53] prepared MIP gels by using bovine serum albumin (BSA) as a template and through graft copolymerization of acrylamide with MBA on chitosan in aqueous medium. They presented the radical induced graft copolymerization mechanism of acrylamide (AAm) to chitosan in the presence of MBA. The resultant MIP gels based on chitosan-*g*-polyacrylamide (CS-*g*-PAM) showed significantly higher imprinting efficiency than those only consisting of polyacrylamide (PAM), as well as better than those composed of chitosan/PAM semi-interpenetrating polymer network (CS/PAM-s-IPN). MIP gels formed with the optimized recipe showed comparatively large rebinding capacity (over 30 mg/g wet gels) and distinct template specificity. Guo *et al.* [54] prepared a hemoglobin-imprinted polymer with acrylamide as functional monomer by grafting of a selective soft polyacrylamide gel to maleic anhydride modified-chitosan beads, inducing the diffusion of the monomers and protein into the pores of the chemically-modified chitosan matrix prior to starting the polymerization. Infrared spectroscopy spectra indicated that acrylamide could be grafted to the maleic anhydride modified chitosan successfully. The MIP exhibited higher adsorption capacity and selectivity toward hemoglobin than the non-imprinted polymer. Moreover, the MIP can be used to remove the protein from different solutions, imparting potential to the polymer for use in biosensor material application. Xia *et al.* [55] reported a semi-interpenetrating polymer network (semi-IPN) hydrogel, which was produced by using the molecularly-imprinted technique to imprint hemoglobin with chitosan and acrylamide as the multiple functional monomers and MBA as the cross-linking agent. The adsorption capacity in the hemoglobin-imprinted semi-IPN hydrogel reached a high value of 36 mg hemoglobin per gram of wet hydrogel, which was considerably higher than that of the non-imprinted semi-IPN hydrogel with the same chemical composition.

### 3.5. Acids

**Figure 9:**
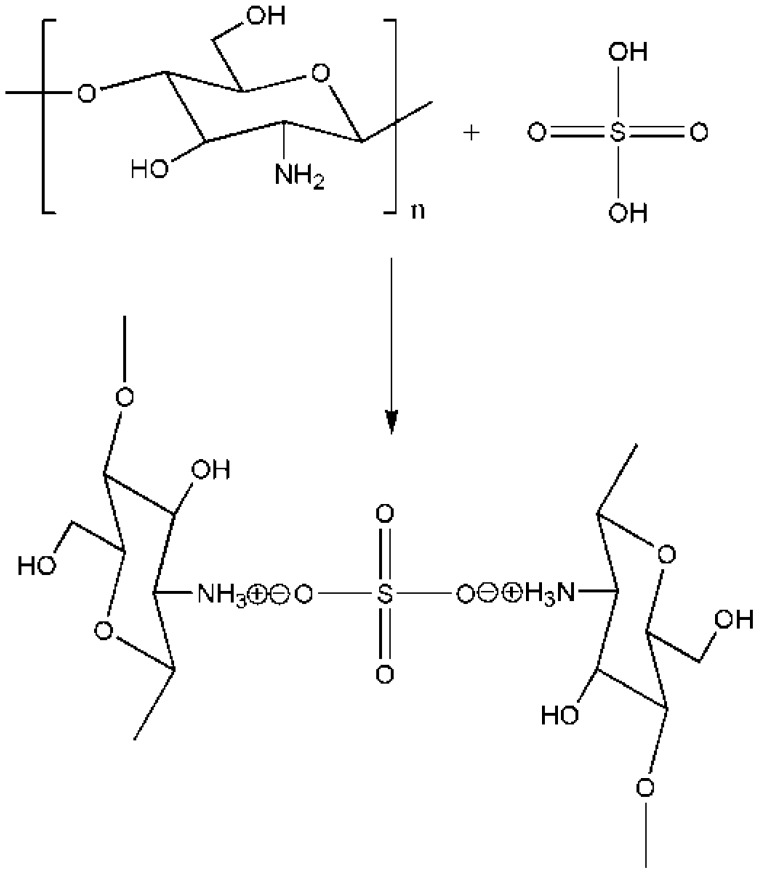
Cross-linking mechanism of chitosan and sulfuric acid.

Aside from the aforementioned chemical cross-linking mechanism, another important mechanism called ionic cross-linking mechanism uses sulfuric acid or tripolyphosphate. Chitosan membranes cross-linked with sulfuric acid have been studied as materials for proton exchange and pervaporation. In the pervaporation of water-alcohol mixtures, sulfuric acid cross-linked chitosan membranes exhibited the highest selectivity with a low permeation flux [56,57,58]. The cross-linking mechanism of chitosan and sulfuric acid is presented in Figure 9.

Nong *et al.* [59] synthesized a l-tryptophane molecularly-imprinted chitosan film using l-tryptophane as a template and chitosan as a film material by alkali treatment with phase inversion and vitriol cross-linkage post-treatment. Fourier transform infrared analysis results showed that chitosan cross-linked with sulfuric acid through ionic bonds, which could prevent its swelling and maintain the imprinting structure well. The l-tryptophane molecularly-imprinted chitosan film showed good permselectivity to the template, whereas *m*(chitosan):*m*(l-tryptophane) = 10:1 and could be applied in the separation of the chiral enantiomer of the amino acid. Wu *et al.* [60] also prepared molecularly-imprinted chitosan membranes through the same method by using l-phenylalanine as an imprinting molecule and evaluated the chiral separation ability of chitosan molecularly-imprinted membranes toward the d- and l-phenylalanine aqueous mixture by permeation experiments. The result shows that improved selectivity with a separation factor (d/l) of 1.43 compared with the control chitosan membrane.

**Table 1:** Application of chitosan in MIT.

Classification	Cross-Linker	Template	Adsorption Capacity	Polymerization Method	Reference
aldehyde	glyoxal	l-aspartic acid	48 ± 0.70 mg/g	sol-gel method	[30]
	glutaraldehyde	As^3+^	6.18 mg/g	emulsion polymerization	[31]
	glutaraldehyde	Cd^2+^	20.70 mg/g	ultraviolet-initiated polymerization	[61]
	glutaraldehyde	bilirubin	8.70 mg/g	inverse phase suspension	[62]
	glutaraldehyde	l-Glutamic acid	42 ± 0.80 mg/g	sol-gel method	[63]
heterocyclic	ECH	Cr^6+^	51 mg/g	precipitation polymerization	[18]
	ECH	Ag^+^	199.20 mg/g	surface imprinting	[39]
	ECH	Co^2+^	92.20 μmol/g	precipitation polymerization	[64]
	ECH	Ag^+^	4.93 mmol/g	surface imprinting	[65]
	ECH	Pb^2+^	139.60 mg/g	surface imprinting	[66]
	ECH	Cu^2+^	21.80 mg/g	precipitation polymerization	[67]
	ECH	Ni^2+^	26 mg/g	precipitation polymerization	[67]
	ECH	Zn^2+^	20.30 mg/g	precipitation polymerization	[67]
	ECH	Ni^2+^	27.39 mg/g	sol-gel method	[68]
	ECH	Ni^2+^	2.75 mmol/g	precipitation polymerization	[69]
	ECH	Hg^2+^	9.02 mg/g	suspension polymerization	[70]
	ECH	perfluorooctane sulfonate	560 μmol/g	precipitation polymerization	[40]
	ECH	alizarin red	40.12 mg/g	surface imprinting	[71]
	ECH	urea	9.61 mg/g	surface imprinting	[72]
	genipin	*O*-xylene	103.30 mg/g	sol-gel method	[46]
ester	EGD	Cu^2+^	35.20 mg/g	surface imprinting	[73]
	EGD	carbamazepine	450 μmol/g	precipitation polymerization	[74]
ether	EGDE	uranyl ion	132 mg/g	sol-gel method	[52]
amide	MBA	bovine serum albumin	39.49 mg/g	bulk polymerization	[53]
	MBA	hemoglobin	35.70 mg/g	bulk polymerization	[54]
	MBA	hemoglobin	36.53 mg/g	sol-gel method	[55]
	MBA	lysozyme	129.80 ± 1.20 mg/g	surface imprinting	[75]
	MBA	ovalbumin	22.94 mg/g	sol-gel method	[76]
Acid	sulfuric acid	l-tryptophane	-	phase inversion	[59]
	sulfuric acid	l-phenylalanine	-	phase inversion	[60]

EGD: Ethylene Glycol Dimethacrylate.

## 4. Conclusions

The current status and highlighted applications of cross-linkers in chitosan MIPs have been described in this review. Although cross-linkers perform an important function in the synthesis of chitosan MIPs, we have to emphasize that cross-linkers are not the sole factor. Polymerization reaction is known as a highly complex process, which could be affected by various other factors, including concentration of chitosan, initiator, temperature and time of polymerization, and the volume of the polymerization mixture. To obtain ideal chitosan MIPs, a variety of factors should be optimized, and considerable work has been conducted to address the problems that hinder the development of chitosan in MIT during past years. In recent years, owing to high selectivity, high sensitivity, low cost, and ease of preparation, chitosan MIPs have become promising tools in the absorption of environmental pollutants, particular heavy metal ions, separation, and the identification of protein and chiral compounds in various media. Moreover, these MIPs could be used in medicine for the treatment of certain diseases. More importantly, the unique properties, such as nontoxicity, biodegradability, biocompatibility, bioactivity, and advantageous physical and mechanical performances make chitosan a promising alternative to other functional monomers and perform an increasingly important function in MIT.

To date, despite remarkable achievements being attained in MIT, substantial development challenges and opportunities still exist. We attempt to tackle several important exploration initiatives to design and synthesize new eco-friendly chitosan MIPs with nontoxic cross-linkers, nontoxic solvent and nontoxic initiator. All involved are nontoxic, thus broadening the application field of MIT in pharmaceutical and food fields. At present, in the progress of promoting green chemistry, we must engage green chemistry with MIT and introduce the idea of green molecular imprinting technology (GMIT), consider and explore GMIT, and then conduct safety evaluations of MIPs prior to use in pharmaceutical and food fields. From this perspective, genipin may be the best choice among cross-linkers. In our subsequent studies, we will exploit MIPs based on chitosan, genipin, and other nontoxic reagents and generalize the initiatives of GMIT so as to successfully broaden the developments of MIPs in pharmaceutical and food fields.
